# Supplementary material for: Anthropogenic ecosystem disturbance and the recovery debt
Source: Nat Commun. 2017 Jan 20;8:14163. doi: 10.1038/ncomms14163 (PMC5263871; doi:10.1038/ncomms14163)
Supplement: Supplementary Information — Supplementary Figures 1-7, Supplementary Tables 1-3 and Supplementary References [file ncomms14163-s1.pdf]

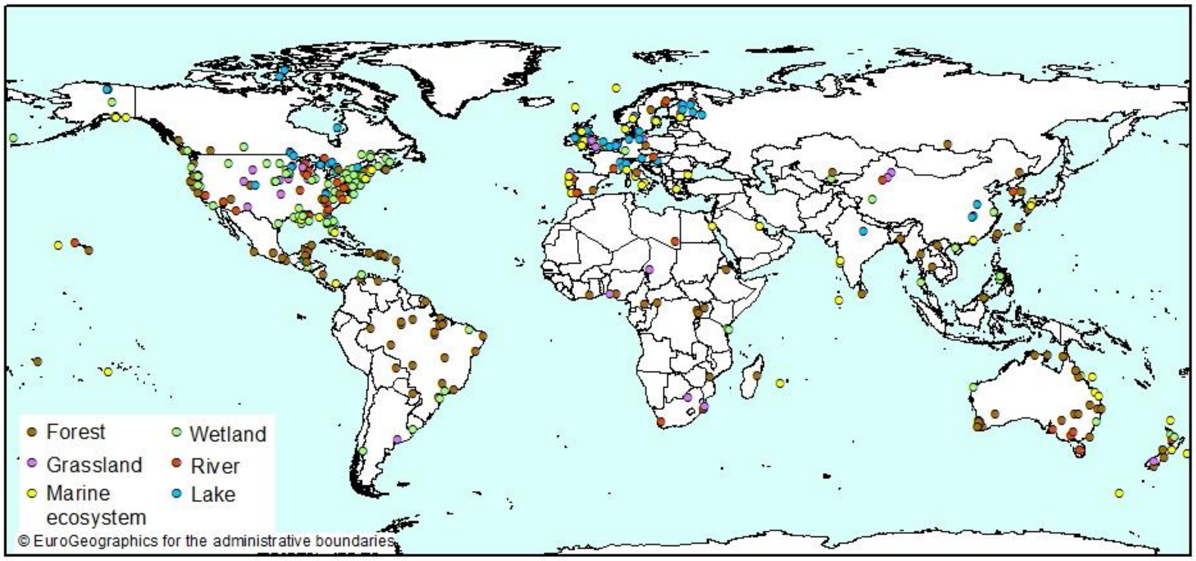

Supplementary Figure 1. Global distribution of the sampling sites per ecosystem category. One dot is used per primary study and each dot may represent several studies and/or sites undergoing recovery.

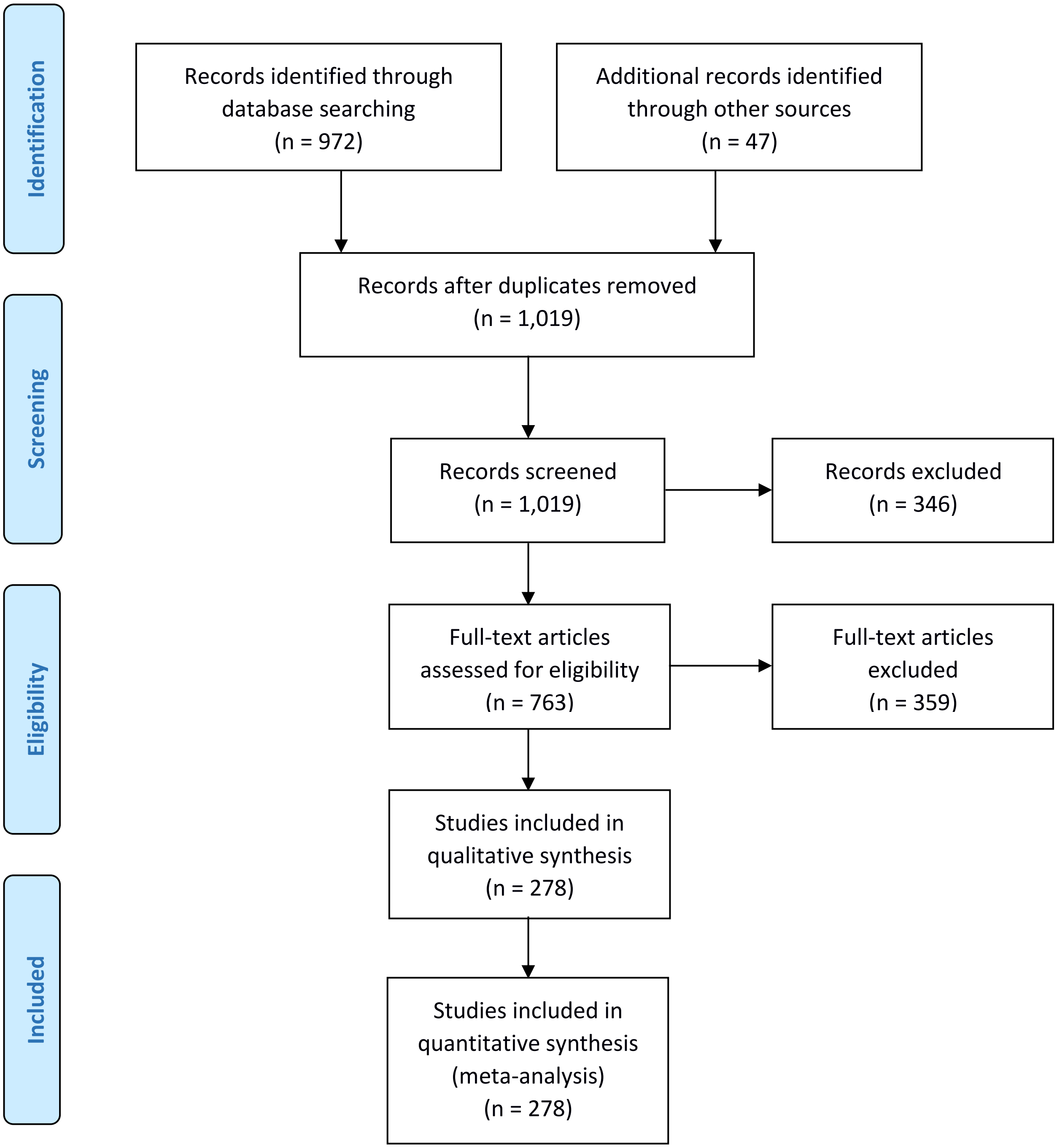

Supplementary Figure 2. PRISMA flow chart of studies included in the meta-analysis. It does not include studies coming from studies refs. 1 and 2 that have been previously published. Structure and template for flow chart from ref. 3.

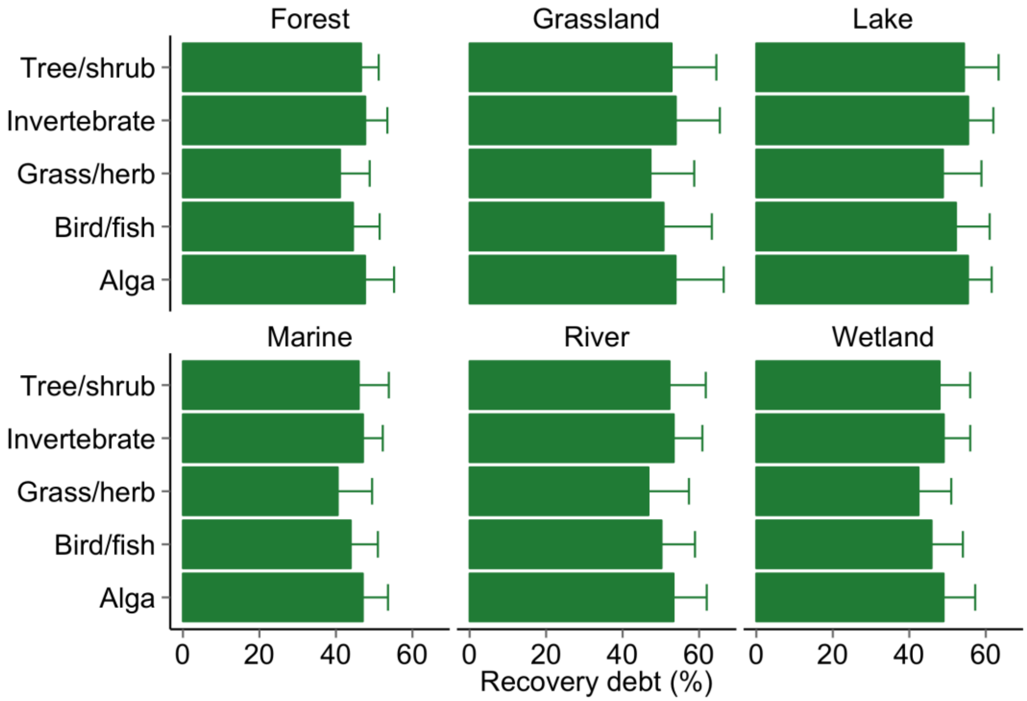

Supplementary Figure 3. Mean recovery debt values and 95% confidence intervals of the abundance of organisms across organism and ecosystem types. Only abundance was included because it was the only metric with enough data to perform the comparison.

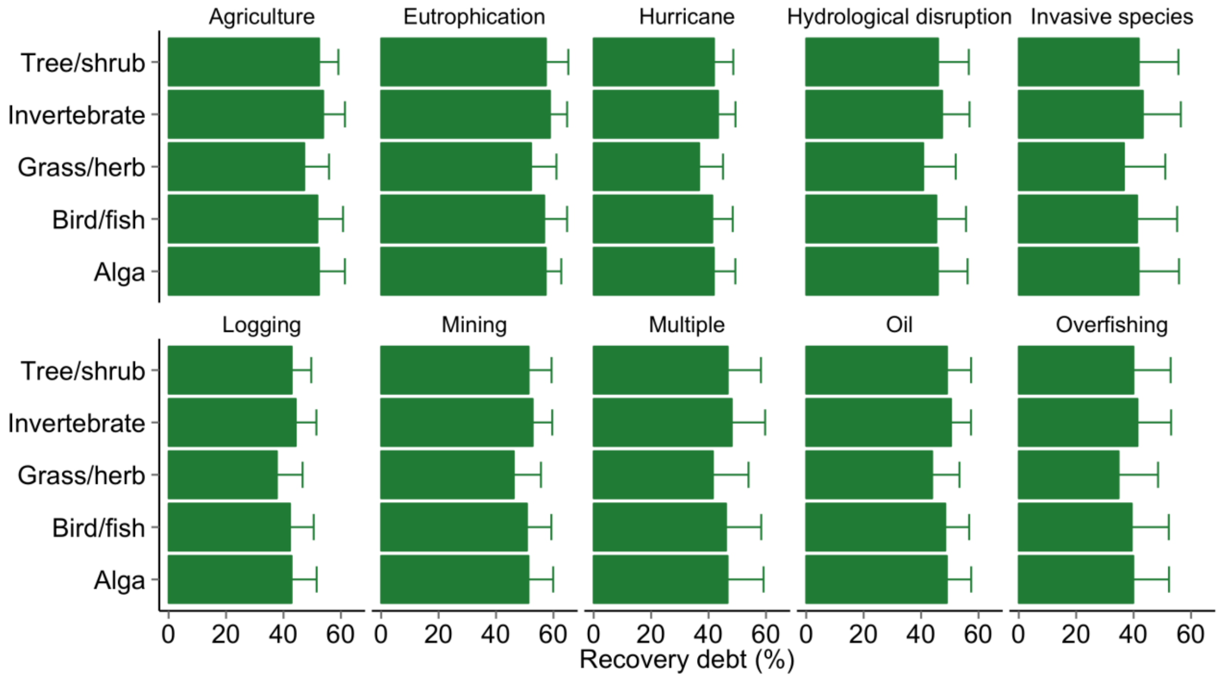

Supplementary Figure 4. Mean recovery debt values and 95% confidence intervals of the abundance of organisms across organism types and degradation categories. Only abundance was included because it was the only metric with enough data to perform the comparison.

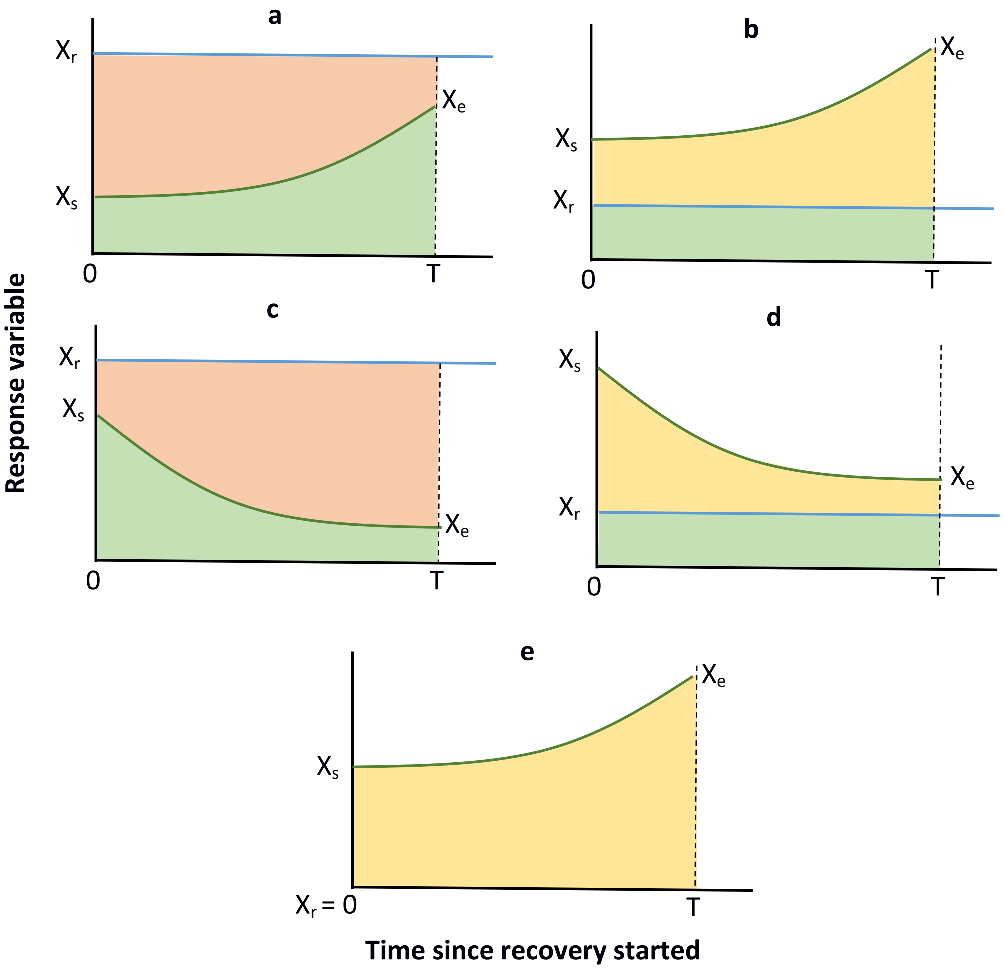

Supplementary Figure 5. Scenarios used to estimate the recovery debt. X_s, value of the outcome measure at the starting point; X_e, value at the end point in the recovery trajectory; and X_r, reference value at the reference system. T is the time elapsed between the starting and the end point. Orange shading represents recovery debt estimated without transformation; yellow shading represents recovery debt estimated after transforming X_s and X_e into $Z_{s,e}$ (see Methods); and green shading represents areas under the curve used to estimate recovery debt. Note than in scenario e, $X_r = 0$.

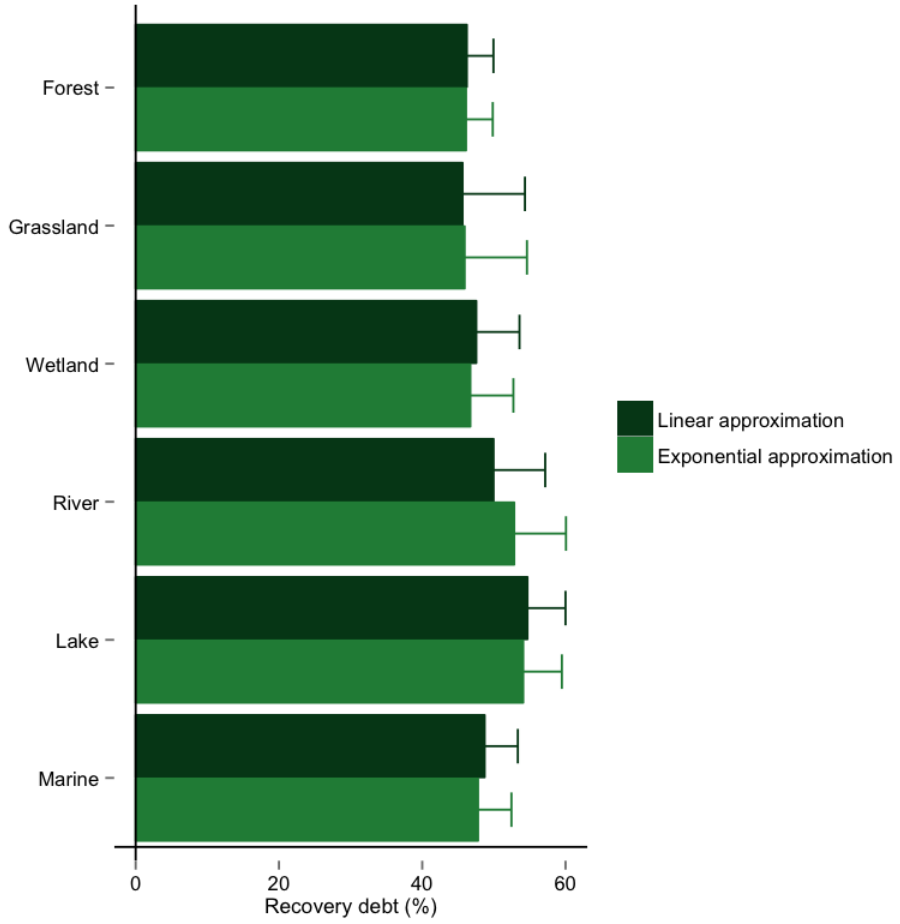

Supplementary Figure 6. Comparison between exponential and linear approaches to estimate the recovery debt. Only abundance means predicted by the model and confidence intervals by ecosystem categories are showed.

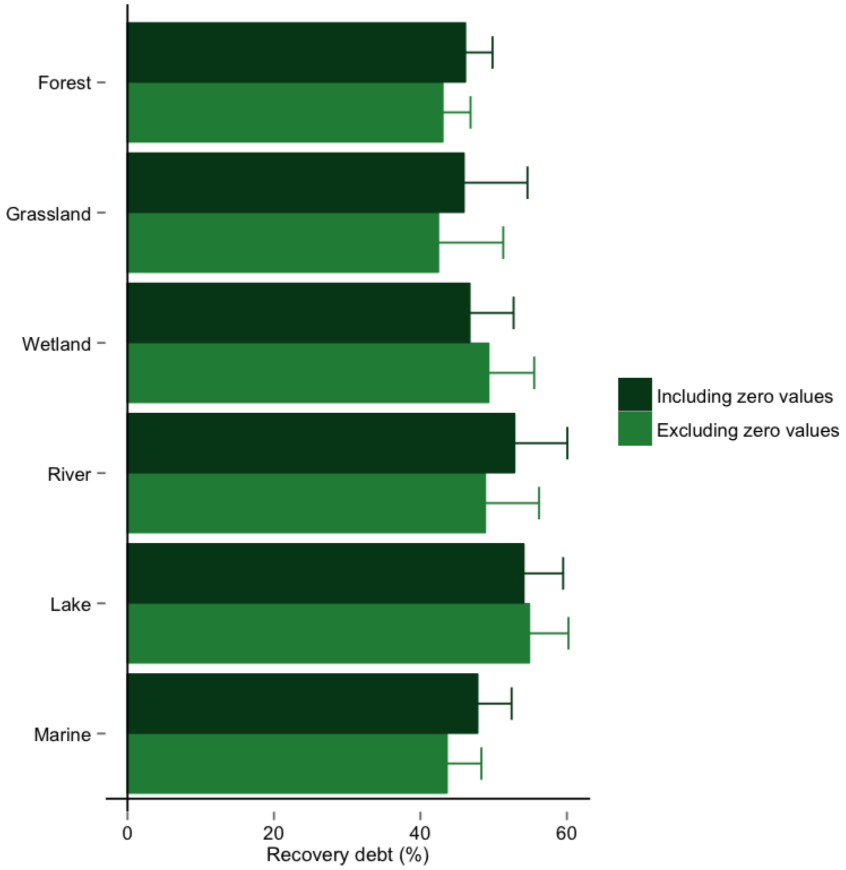

Supplementary Figure 7. Comparison between recovery debts calculated excluding outcome measures containing zero values and including transformed zero values. Only abundance means predicted by the model and confidence intervals by ecosystem categories are showed.

Supplementary Table 1. Distribution of outcome measures, primary studies, sites, and recovering area by ecosystem and disturbance categories. Studies may report outcome measures from multiple ecosystem categories or disturbance categories and thus the totals do not match the total amount of studies selected for the meta-analysis.

Ecosystem	No. outcome measures	Average (min. – max.) no. outcome measures per study	No. studies	No. sites recovering	No. reference sites	Area recovering (km^2)	Studies reporting restored area (%)
Forest	1,616	11.6 (1 – 72)	139	1,334	729	206,010	72
Grassland	254	9.8 (1 – 52)	26	151	53	1,051	59
Wetland	322	7.3 (1 – 32)	44	352	211	15,574	83
River	271	7.7 (1 – 38)	35	156	82	5,340	35
Lake	646	12.2 (1 – 75)	53	353	188	34,823	81
Marine system	707	10.6 (1 – 48)	67	689	349	287,888	65
**Disturbance**							
Agriculture	625	11.2 (1 – 56)	56	525	184	103,180	68
Logging	506	9.7 (1 – 52)	52	368	268	51,017	72
Mining	646	13.5 (1 – 48)	48	274	199	1,320	68
Invasive species	72	8.0 (1 – 9)	9	46	37	24,153	89
Hydrological disruption	123	5.3 (1 – 23)	23	227	93	5,556	64
Eutrophication	811	12.9 (1 – 63)	63	406	214	9,690	78
Oil spill	307	8.3 (1 – 37)	37	148	80	35,244	40
Overfishing	84	6.5 (1 – 13)	13	193	34	325	85
Multiple	58	3.6 (1 – 16)	16	305	72	405,321	100
Hurricanes	584	14.2 (1 – 41)	41	543	414	15,986	67

Supplementary Table 2. Results of the test of moderator effects on the selected response metrics.

Subset	Moderator	Q_M	df	Test of moderators *p*-value
Abundance	Habitat	189.11	5	<0.0001
Abundance	Disturbance	42.83	9	<0.0001
Diversity	Habitat	7.36	5	0.1951
Diversity	Disturbance	21.39	7	0.0032
Carbon	Habitat	5.85	5	0.3213
Carbon	Disturbance	59.35	4	<0.0001
Nitrogen	Habitat	8.21	4	0.084
Nitrogen	Disturbance	9.77	5	0.0822

Supplementary Table 3. Results of the test to select the optimal amount to be added to outcome measures with zero values. We used Mann-Whitney rank sum tests to compare values of the *r* parameter (see Methods) of the database excluding outcome measures that contain zero values and of the full database using nine different strategies. The first row shows the results for the database excluding outcome measures containing zero values. = OM, amount added of the same order of magnitude that X_s and X_g. OM+1, amount added one order of magnitude larger than X_s and X_g. X.1, amount added is the smallest value of the order of magnitude (e.g. 0.1, 1, 10). X.5, amount added is the median of the order of magnitude (e.g. 0.5, 5, 50).

Amount added	n	Median	CI 25%	CI 75%	*U*	*p*
0	3,405	0.0336	0.00212	0.173		
0.01	366	0.317	0.138	1.024	270,049	<0.001
0.05	366	0.217	0.0956	0.665	320,288	<0.001
0.1	366	0.176	0.0743	0.596	345,922	<0.001
0.5	366	0.107	0.0294	0.421	431,846	<0.001
1	366	0.0754	0.0174	0.339	477,439	<0.001
= OM, X.1	366	0.077	0.0322	0.25	446,684	<0.001
= OM, X.5	366	0.0228	0.00733	0.0741	602,144	0.316
OM + 1, X.1	366	0.016	0.00567	0.0538	570,178	0.007
OM + 1, X.5	366	0.00358	0.00126	0.0118	417,130	<0.001

Supplementary references

List of references used in the meta-analysis.

1. Abbott, I., Liddelow, G., Vellios, C., Mellican, A. & Williams, M. Monitoring bird populations after logging in forests of south-west Western Australia: an update from two long-term experimental research case studies. *Conserv. Sci. West. Aust.* **7,** 301–347 (2009).
2. Abbott, I., Burbidge, T., Strehlow, K., Mellican, A. & Wills, A. Logging and burning impacts on cockroaches, crickets and grasshoppers, and spiders in Jarrah forest, Western Australia. *For. Ecol. Manage.* **174,** 383–399 (2003).
3. Adi, S. M., Coleman, D. C. & Read, F. Slow recovery of soil biodiversity in sandy loam soils of Georgia after 25 years of no-tillage management. *Agric. Ecosyst. Environ.* **114,** 323–334 (2006).
4. Adjeroud, M. *et al.* Recurrent disturbances, recovery trajectories, and resilience of coral assemblages on a South Central Pacific reef. *Coral Reefs* **28,** 775–780 (2009).
5. Adum, G. B., Eichhorn, M. P., Oduro, W., Ofori-Boateng, C. & Rodel, M.-O. Two- Stage Recovery of Amphibian Assemblages Following Selective Logging of Tropical Forests. *Conserv. Biol.* **27,** 354–363 (2013).
6. Aide, T. M., Zimmerman, J. K., Pascarella, J. B., Rivera, L. & Marcano-Vega, H. Forest regeneration in a chronosequence of tropical abandoned pastures: Implications for restoration ecology. *Restor. Ecol.* **8,** 328–338 (2000).
7. Aide, T. M., Zimmerman, J. K., Rosario, M. & Marcano, H. Forest recovery in abandoned cattle pastures along an elevational gradient in northeastern Puerto Rico. *Biotropica* **28,** 537–548 (1996).
8. Andersen, A. N., Ludwig, J. A., Lowe, L. M. & Rentz, D. C. F. Grasshopper biodiversity and bioindicators in Australian tropical savannas: responses to disturbance in Kakadu National Park. *Austral Ecol.* **26,** 213–222 (2001).
9. Andersen, D. C. & Nelson, S. M. Rodent use of anthropogenic and ‘natural’desert riparian habitat, lower Colorado River, Arizona. *Regul. Rivers Res. Manag.* **15,** 377–393 (1999).
10. Anton, A., Cebrian, J., Duarte, C. M., Heck Jr., K. L. & Goff, J. Low Impact of Hurricane Katrina on Seagrass Community Structure and Functioning in the Northern Gulf of Mexico. *Bulletin of Marine Science* **85,** 45–59 (2009).
11. Antoniades, D. *et al.* Cultural eutrophication, anoxia, and ecosystem recovery in Meretta Lake, High Arctic Canada. *Limnol. Oceanogr.* **56,** 639–650 (2011).
12. Aravena, J. C., Carmona, M. R., Perez, C. A. & Armesto, J. J. Changes in tree species richness, stand structure and soil properties in a successional chronosequence in northern Chiloe Island, Chile. *Rev. Chil. Hist. Nat.* **75,** 339–360 (2002).
13. Armstrong, K. N. & Nichols, O. G. Long-term trends in avifaunal recolonisation of rehabilitated bauxite mines in the jarrah forest of south-western Australia. *For. Ecol. Manage.* **126,** 213–225 (2000).
14. Arthur, M. A., Coltharp, G. B. & Brown, D. L. Effects of best management practices on forest streamwater quality in eastern Kentucky. *J. Am. Water Resour. Assoc.* **34,** 481–495 (1998).
15. Asner, G. P., Keller, M., Pereira, J. R., Zweede, J. C. & Silva, J. N. M. Canopy damage and recovery after selective logging in Amazonia: Field and satellite studies . *Ecol. Appl.* **14,** 280–298 (2004).
16. Badejo, M. A. Acarine populations of forest and fallow plots in Ile-Ife, Nigeria. *Pedobiologia*

(Jena). **39,** 555–559 (1995).
17. Badosa, A., Frisch, D., Arechederra, A., Serrano, L. & Green, A. J. Recovery of zooplankton diversity in a restored Mediterranean temporary marsh in Do??ana National Park (SW Spain). *Hydrobiologia* **654,** 67–82 (2010).
18. Baer, S. G., Kitchen, D. J., Blair, J. M. & Rice, C. W. Changes in ecosystem structure and function along a chronosequence of restored grasslands. *Ecol. Appl.* **12,** 1688–1701 (2002).
19. Baldwin, L. K. & Bradfield, G. E. Resilience of bryophyte communities in regenerating matrix forests after logging in temperate rainforests of coastal British Columbia This paper is one of a selection of papers published as part of the special Schofield Gedenkschrift. *Botany* **88,** 297–314 (2010).
20. Banks, A. N. *et al.* The Sea empress oil spill (Wales, UK): Effects on common scoter Melanitta nigra in Carmarthen Bay and status ten years later. *Mar. Pollut. Bull.* **56,** 895–902 (2008).
21. Banner, A. & LePage, P. Long-term recovery of vegetation communities after harvesting in the coastal temperate rainforests of northern British Columbia. *Can. J. For. Res.* **38,** 3098–3111 (2008).
22. Banning, N. C., Grant, C. D., Jones, D. L. & Murphy, D. V. Recovery of soil organic matter, organic matter turnover and nitrogen cycling in a post-mining forest rehabilitation chronosequence. *Soil Biol. Biochem.* **40,** 2021–2031 (2008).
23. Banning, N. C., Lalor, B. M., Cookson, W. R., Grigg, A. H. & Murphy, D. V. Analysis of soil microbial community level physiological profiles in native and post-mining rehabilitation forest: Which substrates discriminate? *Appl. Soil Ecol.* **56,** 27–34 (2012).
24. Banning, N. C., Phillips, I. R., Jones, D. L. & Murphy, D. V. Development of microbial diversity and functional potential in bauxite residue sand under rehabilitation. *Restor. Ecol.* **19,** 78–87 (2011).
25. Barber, W. E., McDonald, L. L., Erickson, W. P. & Vallarino, M. Effect of the Exxon-Valdez oil-spill on intertidal fish - a field-study. *Trans. Am. Fish. Soc.* **124,** 461–476 (1995).
26. Barr, J. G., Engel, V., Smith, T. J. & Fuentes, J. D. Hurricane disturbance and recovery of energy balance, CO 2 fluxes and canopy structure in a mangrove forest of the Florida Everglades. *Agric. For. Meteorol.* **153,** 54–66 (2012).
27. Baustian, J., Mendelssohn, I., Lin, Q. & Rapp, J. In situ burning restores the ecological function and structure of an oil-impacted coastal marsh. *Environ. Manage.* **46,** 781–789 (2010).
28. Belote, R. T., Jones, R. H. & Wieboldt, T. F. Compositional stability and diversity of vascular plant communities following logging disturbance in Appalachian forests. *Ecol. Appl.* **22,** 502–516 (2012).
29. Benstead, J. P. *et al.* Recovery of three arctic stream reaches from experimental nutrient enrichment. *Freshw. Biol.* **52,** 1077–1089 (2007).
30. Bevilacqua, S., Terlizzi, A., Fraschetti, S., Russo, G. F. & Boero, F. Mitigating human disturbance: can protection influence trajectories of recovery in benthic assemblages? *J. Anim. Ecol.* **75,** 908–920 (2006).
31. Bihn, J. H., Verhaagh, M., Braendle, M. & Brandl, R. Do secondary forests act as refuges for old growth forest animals? Recovery of ant diversity in the Atlantic forest of Brazil. *Biol. Conserv.* **141,** 733–743 (2008).
32. Bonne, W. M. I. Macrobenthos characteristics and distribution, following intensive sand extraction from a subtidal sandbank. *J. Coast. Res.* 141–150 (2010).

33. Bonnell, T. R., Reyna-Hurtado, R. & Chapman, C. A. Post-logging recovery time is longer than expected in an East African tropical forest. *For. Ecol. Manage.* **261,** 855–864 (2011).
34. Bosire, J. O., Dahdouh-Guebas, F., Kairo, J. G., Cannicci, S. & Koedam, N. Spatial variations in macrobenthic fauna recolonisation in a tropical mangrove bay. *Biodivers. Conserv.* **13,** 1059–1074 (2004).
35. Boucher, D. H., Vandermeer, J. H., Mallona, M. A., Zamora, N. & Perfecto, I. Resistance and resilience in a directly regenerating rainforest: Nicaraguan trees of the Vochysiaceae after Hurricane Joan. *For. Ecol. Manage.* **68,** 127–136 (1994).
36. Bradley, M., House, A., Robertson, M. & Wild, C. Vegetation succession and recovery of ecological values in the southern Queensland Brigalow Belt. *Ecol. Manag. Restor.* **11,** 113–118 (2010).
37. Bradshaw, E. G. & Anderson, N. J. Validation of a diatom–phosphorus calibration set for Sweden. *Freshw. Biol.* **46,** 1035–1048 (2001).
38. Brady, C. J. & Noske, R. A. Succession in Bird and Plant Communities over a 24- Year Chronosequence of Mine Rehabilitation in the Australian Monsoon Tropics. *Restor. Ecol.* **18,** 855–864 (2010).
39. Bravo-Garza, M. R. & Bryan, R. B. Soil properties along cultivation and fallow time sequences on vertisols in northeastern Mexico. *Soil Sci. Soc. Am. J.* **69,** 473–481 (2005).
40. Broadbent, E. N. *et al.* Recovery of forest structure and spectral properties after selective logging in lowland Bolivia. *Ecol. Appl.* **16,** 1148–1163 (2006).
41. Brooks, C. N. & Merenlender, A. M. Determining the pattern of oak woodland regeneration for a cleared watershed in northwest California: A necessary first step for restoration. *Restor. Ecol.* **9,** 1–12 (2001).
42. Brooks, R. P., Wardrop, D. H., Cole, C. A. & Campbell, D. A. Are we purveyors of wetland homogeneity?: A model of degradation and restoration to improve wetland mitigation performance. *Ecol. Eng.* **24,** 331–340 (2005).
43. Brosse, S., Grenouillet, G., Gevrey, M., Khazraie, K. & Tudesque, L. Small-scale gold mining erodes fish assemblage structure in small neotropical streams. *Biodivers. Conserv.* **20,** 1013–1026 (2011).
44. Brown, S., Sprenger, M., Maxemchuk, A. & Compton, H. Ecosystem function in alluvial tailings after biosolids and lime addition. *J. Environ. Qual.* **34,** 139–148 (2005).
45. Brye, K. R., Gower, S. T., Norman, J. M. & Bundy, L. G. Carbon budgets for a prairie and agroecosystems: effects of land use and interannual variability. *Ecol. Appl.* **12,** 962–979 (2002).
46. Burkholder, J. M., Tomasko, D. A. & Touchette, B. W. Seagrasses and eutrophication. *J. Exp. Mar. Bio. Ecol.* **350,** 46–72 (2007).
47. Bythell, J. C., Hillis-Starr, Z. M. & Rogers, C. S. Local variability but landscape stability in coral reef communities following repeated hurricane impacts. *Mar. Ecol. Prog. Ser.* **204,** 93–100 (2000).
48. Callaham, M. A., Stewart, A. J., Alarcón, C. & McMillen, S. J. Effects of earthworm (Eisenia fetida) and wheat (Triticum aestivum) straw additions on selected properties of petroleum-contaminated soils. *Environ. Toxicol. Chem.* **21,** 1658–1663 (2002).
49. Cardoso, P. G., Raffaelli, D., Lillebø, A. I., Verdelhos, T. & Pardal, M. A. The impact of extreme flooding events and anthropogenic stressors on the macrobenthic communities' dynamics. *Estuar. Coast. Shelf Sci.* **76,** 553–565 (2008).
50. Carey, A. B., Peterson, C. & Maguire, D. A. Active intentional management (AIM) for

biodiversity and other forest values. *Gen. Tech. Report-Pacific Northwest Res. Station. USDA For. Serv.* 227–234 (2005).
51. Carstensen, J., Krause-Jensen, D., Markager, S., Timmermann, K. & Windolf, J. Water clarity and eelgrass responses to nitrogen reductions in the eutrophic Skive Fjord, Denmark. *Hydrobiologia* **704,** 293–309 (2013).
52. Carter, D. L. & Blair, J. M. Recovery of Native Plant Community Characteristics on a Chronosequence of Restored Prairies Seeded into Pastures in West- Central Iowa. *Restor. Ecol.* **20,** 170–179 (2012).
53. Carvalho, L. *et al.* Water quality of Loch Leven: responses to enrichment, restoration and climate change. *Hydrobiologia* **681,** 35–47 (2012).
54. Casper, A. F., Thorp, J. H., Davies, S. P. & Courtemanch, D. L. Ecological responses of zoobenthos to dam removal on the Kennebec River, Maine, USA. *Large Rivers* **16,** 541–555 (2006).
55. Catalano, M. J., Bozek, M. A. & Pellett, T. D. Effects of dam removal on fish assemblage structure and spatial distributions in the Baraboo River, Wisconsin. *North Am. J. Fish. Manag.* **27,** 519–530 (2007).
56. Cebrian, J. *et al.* The impact of Hurricane Ivan on the primary productivity and metabolism of marsh tidal creeks in the NorthCentral Gulf of Mexico. *Aquat. Ecol.* **42,** 391–404 (2008).
57. Champeau, T. R., Stevens, P. W. & Blewett, D. A. Comparison of Fish Community Metrics To Assess Long-Term Changes and Hurricane Impacts At Peace River, Florida. *Florida Sci.* **72,** 289–309 (2009).
58. Chapman, C. A. & Chapman, L. J. Forest regeneration in logged and unlogged forests of Kibale National Park, Uganda. *Biotropica* **29,** 396–412 (1997).
59. Cheal, A. J. *et al.* Coral-macroalgal phase shifts or reef resilience: Links with diversity and functional roles of herbivorous fishes on the Great Barrier Reef. *Coral Reefs* **29,** 1005–1015 (2010).
60. Chen, L. H., Chu, K. C. M. & Chiu, Y. W. Impacts of natural disturbance on fish communities in the Tachia River, Taiwan. *Hydrobiologia* **522,** 149–164 (2004).
61. Chinea, J. D. Tropical forest succession on abandoned farms in the Humacao Municipality of eastern Puerto Rico. *For. Ecol. Manage.* **167,** 195–207 (2002).
62. Chung, I. Y. *et al.* Effects of oil spill on seawater infiltration and macrobenthic community in tidal flats. *Mar. Pollut. Bull.* **49,** 959–963 (2004).
63. Clark, C. M., Hobbie, S. E., Venterea, R. & Tilman, D. Long- lasting effects on nitrogen cycling 12 years after treatments cease despite minimal long- term nitrogen retention. *Glob. Chang. Biol.* **15,** 1755–1766 (2009).
64. Clark, S. & Edwards, A. J. Use of artificial reef structures to rehabilitate reef flats degraded by coral mining in the Maldives. *Bull. Mar. Sci.* **55,** 724–744 (1994).
65. Clarke, P. J. & Ward, T. J. The response of southern hemisphere saltmarsh plants and gastropods to experimental contamination by petroleum hydrocarbons. *J. Exp. Mar. Bio. Ecol.* **175,** 43–57 (1994).
66. Colón, S. M. & Lugo, A. E. Recovery of a Subtropical Dry Forest After Abandonment of Different Land Uses1. *Biotropica* **38,** 354–364 (2006).
67. Constantine, J. A., Pasternack, G. B. & Johnson, M. L. Logging effects on sediment flux observed in a pollen-based record of overbank deposition in a northern California catchment. *Earth Surf. Process. Landforms* **30,** 813–821 (2005).
68. Constantino, R. *et al.* Clam dredging effects and subsequent recovery of benthic communities

at different depth ranges. *Mar. Environ. Res.* **67,** 89–99 (2009).
69. Cooper-ellis, A. S., Foster, D. R., Carlton, G. & Lezberg, A. Forest Response to Catastrophic Wind : Results from an Experimental Hurricane. *Ecology* **80,** 2683–2696 (2010).
70. Coops, H., Tockner, K., Amoros, C., Hein, T. & Quinn, G. in *Wetlands and natural resource management* 15–32 (Springer, 2006).
71. Covich, A. P. *et al.* Post-Hurricane Hugo Increases in Atyid Shrimp Abundances in a Puerto Rican Montane Stream. *Biotropica* **23,** 448–454 (2011).
72. Crawford, J. A. & Semlitsch, R. D. Post-disturbance effects of even-aged timber harvest on stream salamanders in Southern Appalachian forests. *Anim. Conserv.* **11,** 369–376 (2008).
73. Crona, B. I. & Rönnbäck, P. Community structure and temporal variability of juvenile fish assemblages in natural and replanted mangroves, Sonneratia alba Sm., of Gazi Bay, Kenya. *Estuar. Coast. Shelf Sci.* **74,** 44–52 (2007).
74. Cronin, G., Lewis, W. M. & Schiehser, M. A. Influence of freshwater macrophytes on the littoral ecosystem structure and function of a young Colorado reservoir. *Aquat. Bot.* **85,** 37–43 (2006).
75. Crossetti, L. O. & Bicudo, C. E. de M. Structural and functional phytoplankton responses to nutrient impoverishment in mesocosms placed in a shallow eutrophic reservoir (Garças Pond), São Paulo, Brazil. *Hydrobiologia* **541,** 71–85 (2005).
76. Cuevas, Y. A. & Zalba, S. M. Recovery of Native Grasslands after Removing Invasive Pines. *Restor. Ecol.* **18,** 711–719 (2010).
77. da Silva, D. K. A. *et al.* Diversity of arbuscular mycorrhizal fungi in restinga and dunes areas in Brazilian Northeast. *Biodivers. Conserv.* **21,** 2361–2373 (2012).
78. Daïnou, K. *et al.* Soil seed bank characteristics in Cameroonian rainforests and implications for post-logging forest recovery. *Ecol. Eng.* **37,** 1499–1506 (2011).
79. Dangi, S. R., Stahl, P. D., Wick, A. F., Ingram, L. J. & Buyer, J. S. Soil microbial community recovery in reclaimed soils on a surface coal mine site. *Soil Sci. Soc. Am. J.* **76,** 915–924 (2012).
80. Davidson, E. a *et al.* Recuperation of nitrogen cycling in Amazonian forests following agricultural abandonment. *Nature* **447,** 995–8 (2007).
81. Davies, P. E. & Nelson, M. The effect of steep slope logging on fine sediment infiltration into the beds of ephemeral and perennial streams of the Dazzler Range, Tasmania, Australia. *J. Hydrol.* **150,** 481–504 (1993).
82. Dawe, N. K., Bradfield, G. E., Boyd, W. S., Trethewey, D. E. C. & Zolbrod, A. N. Marsh creation in a northern Pacific estuary: Is thirteen years of monitoring vegetation dynamics enough? *Conserv. Ecol.* **4,** (2000).
83. de Camargo, P. B. *et al.* Soil carbon dynamics in regrowing forest of eastern Amazonia. *Glob. Chang. Biol.* **5,** 693–702 (1999).
84. Dean, T. A. & Jewett, S. C. Habitat-specific recovery of shallow subtidal communities following the Exxon Valdez oil spill. *Ecol. Appl.* **11,** 1456–1471 (2001).
85. De Biasi, A. M. Impact of experimental trawling on the benthic assemblage along the Tuscany coast (north Tyrrhenian Sea, Italy). *ICES J. Mar. Sci.* **61,** 1260–1266 (2004).
86. Descheemaeker, K. *et al.* Runoff on slopes with restoring vegetation: A case study from the Tigray highlands, Ethiopia. *J. Hydrol.* **331,** 219–241 (2006).
87. Dick, T. M., Streever, W. J. & Osunkoya, O. O. Decomposition of Sarcocornia quinqueflora on an Iron- Smelting Slag Substrate. *Restor. Ecol.* **10,** 11–15 (2002).
88. Doi, R. & Ranamukhaarachchi, S. L. Community-level Physiological Profiling in Monitoring

Rehabilitative Effects of Acacia auriculiformis Plantation on Degraded Land in Sakaerat, Thailand. *Silva Fenn.* **43,** 739–754 (2009).
89. Dokulil, M. T. & Teubner, K. Do phytoplankton communities correctly track trophic changes? An assessment using directly measured and palaeolimnological data. *Freshw. Biol.* **50,** 1594–1604 (2005).
90. Dominguez- Haydar, Y. & Armbrecht, I. Response of ants and their seed removal in rehabilitation areas and forests at El Cerrejon coal mine in Colombia. *Restor. Ecol.* **19,** 178–184 (2011).
91. Donath, T. W., Hölzel, N. & Otte, A. Influence of competition by sown grass, disturbance and litter on recruitment of rare flood-meadow species. *Biol. Conserv.* **130,** 315–323 (2006).
92. Donohue, I. *et al.* Rapid ecosystem recovery from diffuse pollution after the Great Irish Famine. *Ecol. Appl.* **20,** 1733–1743 (2010).
93. Duchrow, R. M. Effects of barite tailings on benthos and turbidity of two Ozark streams. *Trans. Missouri Acad. Sci.* **16,** 55–66 (1982).
94. Dudley, B. *et al.* Changes in aquatic macrophyte communities in Loch Leven: evidence of recovery from eutrophication? *Hydrobiologia* **681,** 49–57 (2012).
95. Duffy, D. C. & Meier, A. J. Do Appalachian herbaceous understories ever recover from clearcutting? *Conserv. Biol.* **6,** 196–201 (1992).
96. Eaton, J. M. & Lawrence, D. Loss of carbon sequestration potential after several decades of shifting cultivation in the Southern Yucat·n. *For. Ecol. Manage.* **258,** 949–958 (2009).
97. Ebrahimnezhad, M. & Harper, D. M. The biological effectiveness of artificial riffles in river rehabilitation. *Aquat. Conserv. Mar. Freshw. Ecosyst.* **7,** 187–197 (1997).
98. Edmondson, J., Terribile, E., Carroll, J. A., Price, E. A. C. & Caporn, S. J. M. The legacy of nitrogen pollution in heather moorlands: Ecosystem response to simulated decline in nitrogen deposition over seven years. *Sci. Total Environ.* **444,** 138–144 (2013).
99. Ekdahl, E. J. *et al.* Diatom assemblage response to Iroquoian and Euro-Canadian eutrophication of Crawford Lake, Ontario, Canada. *J. Paleolimnol.* **37,** 233–246 (2007).
100. Ellis, L. M., Molles, M. C. & Crawford, C. S. Influence of experimental flooding on litter dynamics in a Rio Grande riparian forest, New Mexico. *Restor. Ecol.* **7,** 193–204 (1999).
101. Faria, M. S., Lopes, R. J., Malcato, J., Nogueira, A. J. A. & Soares, A. M. V. M. In situ bioassays with Chironomus riparius larvae to biomonitor metal pollution in rivers and to evaluate the efficiency of restoration measures in mine areas. *Environ. Pollut.* **151,** 213–221 (2008).
102. Ferreira, S. M. & Van Aarde, R. J. Maintaining diversity through intermediate disturbances: evidence from rodents colonizing rehabilitating coastal dunes. *Afr. J. Ecol.* **38,** 286–294 (2000).
103. Ferrier-Pages, C., Gattuso, J.-P., Dallot, S. & Jaubert, J. Effect of nutrient enrichment on growth and photosynthesis of the zooxanthellate coral Stylophora pistillata. *Coral Reefs* **19,** 103–113 (2000).
104. Fimbel, R. A. & Fimbel, C. C. The role of exotic conifer plantations in rehabilitating degraded tropical forest lands: a case study from the Kibale Forest in Uganda. *For. Ecol. Manage.* **81,** 215–226 (1996).
105. Finsinger, W., Bigler, C., Krähenbühl, U., Lotter, A. F. & Ammann, B. Human impacts and eutrophication patterns during the past~ 200 years at Lago Grande di Avigliana (N. Italy). *J. Paleolimnol.* **36,** 55–67 (2006).

106. Freeman, A. N. D., Pias, K. & Vinson, M. F. The impact of Tropical Cyclone Larry on bird communities in fragments of the endangered rainforest Type 5b. *Austral Ecol.* **33,** 532–540 (2008).
107. Frenkel, R. E. & Morlan, J. C. Can we restore our salt marshes? Lessons from the Salmon River, Oregon. *Northwest Environ. J.* **7,** 119–135 (1991).
108. Galán, P. Colonization of spoil benches of an opencast lignite mine in Northwest Spain by amphibians and reptiles. *Biol. Conserv.* **79,** 187–195 (1997).
109. Garcia-Regueira, X., Tato, R., Moreira, J. & Urgorri, V. Temporal evolution of polychaete assemblages on intertidal hard substrata at two localities of the Galician coast after the 'prestige'oil spill. *Thalass. An Int. J. Mar. Sci.* **26,** 33–45 (2010).
110. Garrity, S. D. & Levings, S. C. Effects of an oil spill on some organisms living on mangrove (Rhizophora mangle L.) roots in low wave-energy habitats in Caribbean Panama. *Mar. Environ. Res.* **35,** 251–271 (1993).
111. Gehring, C., Denich, M. & Vlek, P. L. G. Resilience of secondary forest regrowth after slash-and-burn agriculture in central Amazonia. *J. Trop. Ecol.* **21,** 519–527 (2005).
112. Glen, M. *et al.* Ectomycorrhizal fungal communities of rehabilitated bauxite mines and adjacent, natural jarrah forest in Western Australia. *For. Ecol. Manage.* **255,** 214–225 (2008).
113. Gonzalez, C., Urrego, L. E., Martinez, J. I., Polania, J. & Yokoyama, Y. Mangrove dynamics in the southwestern Caribbean since the Little Ice Age: a history of human and natural disturbances. *The Holocene* **20,** 849–861 (2010).
114. Gormsen, D., Hedlund, K. & Huifu, W. Diversity of soil mite communities when managing plant communities on set-aside arable land. *Appl. Soil Ecol.* **31,** 147–158 (2006).
115. Gould, S. F. Comparison of Post- mining Rehabilitation with Reference Ecosystems in Monsoonal Eucalypt Woodlands, Northern Australia. *Restor. Ecol.* **20,** 250–259 (2012).
116. Gratton, C. & Denno, R. F. Restoration of arthropod assemblages in a Spartina salt marsh following removal of the invasive plant Phragmites australis. *Restor. Ecol.* **13,** 358–372 (2005).
117. Gray, N. F. & Delaney, E. Comparison of benthic macroinvertebrate indices for the assessment of the impact of acid mine drainage on an Irish river below an abandoned Cu–S mine. *Environ. Pollut.* **155,** 31–40 (2008).
118. Greenfield, B. K. *et al.* Mechanical Shredding of Water Hyacinth (Eichhornia crassipes): Effects on Water Quality in the Sacramento-San Joaquin River Delta, California. *Estuaries Coasts* **30,** 627–640 (2007).
119. Greening, H. & Janicki, A. Toward reversal of eutrophic conditions in a subtropical estuary: Water quality and seagrass response to nitrogen loading reductions in Tampa Bay, Florida, USA. *Environ. Manage.* **38,** 163–178 (2006).
120. Groffman, P. M., Dorsey, A. M. & Mayer, P. M. N processing within geomorphic structures in urban streams. *J. North Am. Benthol. Soc.* **24,** 613–625 (2005).
121. Grogan, J. *et al.* What loggers leave behind: impacts on big-leaf mahogany (Swietenia macrophylla) commercial populations and potential for post-logging recovery in the Brazilian Amazon. *For. Ecol. Manage.* **255,** 269–281 (2008).
122. Guillemot, N., Chabanet, P. & Le Pape, O. Cyclone effects on coral reef habitats in New Caledonia (South Pacific). *Coral Reefs* **29,** 445–453 (2010).
123. Gunn, J., Sarrazin-Delay, C., Wesolek, B., Stasko, A. & Szkokan-Emilson, E. Delayed recovery of benthic macroinvertebrate communities in Junction Creek, Sudbury, Ontario,

after the diversion of acid mine drainage. *Hum. Ecol. Risk Assess.* **16,** 901–912 (2010).
124. Guo, L., Li, Z., Xie, P. & Ni, L. Assessment effects of cage culture on nitrogen and phosphorus dynamics in relation to fallowing in a shallow lake in China. *Aquac. Int.* **17,** 229–241 (2009).
125. Hajnal, E. & Padisák, J. Analysis of long-term ecological status of Lake Balaton based on the ALMOBAL phytoplankton database. *Hydrobiologia* **599,** 227–237 (2008).
126. Hall-Spencer 2000
127. Hansen, J. F. & Hayes, D. B. Long- term implications of dam removal for macroinvertebrate communities in michigan and wisconsin rivers, united states. *River Res. Appl.* **28,** 1540–1550 (2012).
128. Harden, C. P. & Mathews, L. Rainfall response of degraded soil following reforestation in the Copper Basin, Tennessee, USA. *Environ. Manage.* **26,** 163–174 (2000).
129. Harig, A. L. & Bain, M. B. Defining and restoring biological integrity in wilderness lakes. *Ecol. Appl.* **8,** 71–87 (1998).
130. Hasselquist, N. J., Santiago, L. S. & Allen, M. F. Belowground nitrogen dynamics in relation to hurricane damage along a tropical dry forest chronosequence. *Biogeochemistry* **98,** 89–100 (2010).
131. Heneghan, L., Salmore, A. & Crossley, D. A. Recovery of decomposition and soil microarthropod communities in an Appalachian watershed two decades after a clearcut. *For. Ecol. Manage.* **189,** 353–362 (2004).
132. Hester, M. W. & Mendelssohn, I. A. Long-term recovery of a Louisiana brackish marsh plant community from oil-spill impact: vegetation response and mitigating effects of marsh surface elevation. *Mar. Environ. Res.* **49,** 233–254 (2000).
133. Hill, N. M., Keddy, P. A. & Wisheu, I. C. A hydrological model for predicting the effects of dams on the shoreline vegetation of lakes and reservoirs. *Environ. Manage.* **22,** 723–736 (1998).
134. Hobaek, A. *et al.* Eutrophication, recovery and temperature in Lake Mjøsa: detecting trends with monitoring data and sediment records. *Freshw. Biol.* **57,** 1998–2014 (2012).
135. Holl, K. D. The effect of coal surface mine reclamation on diurnal lepidopteran conservation. *J. Appl. Ecol.* 225–236 (1996).
136. Howorth, R. T. & Pendry, C. A. Post-cultivation secondary succession in a Venezuelan lower montane rain forest. *Biodivers. Conserv.* **15,** 693–715 (2006).
137. Huang, M. & Asner, G. P. Long-term carbon loss and recovery following selective logging in Amazon forests. *Glob. Biogeochem. Cycles* **24,** GB3028 (2010).
138. Hughes, R. F., Uowolo, A. L. & Togia, T. P. Recovery of native forest after removal of an invasive tree, Falcataria moluccana, in American Samoa. *Biol. Invasions* **14,** 1393–1413 (2012).
139. Hüttl, R. F. & Weber, E. Forest ecosystem development in post-mining landscapes: a case study of the Lusatian lignite district. *Naturwissenschaften* **88,** 322–329 (2001).
140. Hylander, K. & Weibull, H. Do time- lagged extinctions and colonizations change the interpretation of buffer strip effectiveness?–a study of riparian bryophytes in the first decade after logging. *J. Appl. Ecol.* **49,** 1316–1324 (2012).
141. Ibelings, B. W. *et al.* Resilience of alternative stable states during the recovery of shallow lakes from eutrophication: Lake Veluwe as a case study. *Ecosystems* **10,** 4–16 (2007).
142. Imbert, D. & Portecop, J. Hurricane disturbance and forest resilience: Assessing

structural vs. functional changes in a Caribbean dry forest. *For. Ecol. Manage.* **255,** 3494–3501 (2008).
143. Iverson, S. A. & Esler, D. Harlequin Duck population injury and recovery dynamics following the 1989 Exxon Valdez oil spill. *Ecol. Appl.* **20,** 1993–2006 (2010).
144. James, T. & Chimney, M. Hurricane effects on a shallow lake ecosystem, Lake Okeechobee, Florida (USA). *Fundam. Appl. Limnol.* **172,** 273–287 (2008).
145. Jansen, A. Avian use of restoration plantings along a creek linking rainforest patches on the Atherton Tablelands, North Queensland. *Restor. Ecol.* **13,** 275–283 (2005).
146. Jewett, S. C., Dean, T. A., Smith, R. O. & Blanchard, A. 'Exxon Valdez' oil spill: Impacts and recovery in the soft-bottom benthic community in and adjacent to eelgrass beds. *Mar. Ecol. Prog. Ser.* 59–83 (1999).
147. Jiang, J.-G., Wu, S.-G. & Shen, Y.-F. Effects of seasonal succession and water pollution on the protozoan community structure in an eutrophic lake. *Chemosphere* **66,** 523–532 (2007).
148. Jin, K.-R., Chang, N.-B., Ji, Z.-G. & James, R. T. Hurricanes affect the sediment and environment in lake okeechobee. *Crit. Rev. Environ. Sci. Technol.* **41,** (2011).
149. Johnson, A. B. & Winker, K. Short-term hurricane impacts on a neotropical community of marked birds and implications for early- stage community resilience. *PLoS One* **5,** (2010).
150. Jones, H. P. Seabird islands take mere decades to recover following rat eradication. *Ecol. Appl.* **23,** 515–522 (2010).
151. Jones, H. P. Prognosis for ecosystem recovery following rodent eradication and seabird restoration in an island archipelago. *Ecol. Appl.* **20,** 1204–1216 (2010).
152. Jones, D. A., Plaza, J., Watt, I. & Al Sanei, M. Long-term (1991–1995) monitoring of the intertidal biota of Saudi Arabia after the 1991 Gulf War oil spill. *Mar. Pollut. Bull.* **36,** 472–489 (1998).
153. Kaiser, M. Changes in megafaunal benthic communities in different habitats after trawling disturbance. *ICES J. Mar. Sci.* **55,** 353–361 (1998).
154. Kammesheidt, L. Forest recovery by root suckers and above-ground sprouts after slash-and-burn agriculture, fire and logging in Paraguay and Venezuela. *J. Trop. Ecol.* **15,** 143–157 (1999).
155. Kanehl, P. D., Lyons, J. & Nelson, J. E. Changes in the habitat and fish community of the Milwaukee River, Wisconsin, following removal of the Woolen Mills Dam. *North Am. J. Fish. Manag.* **17,** 387–400 (1997).
156. Kang, C.-K. *et al.* Food web structure of a restored macroalgal bed in the eastern Korean peninsula determined by C and N stable isotope analyses. *Mar. Biol.* **153,** 1181–1198 (2008).
157. Kanowski, J., Winter, J. W. & Catterall, C. P. Impacts of Cyclone Larry on arboreal folivorous marsupials endemic to upland rainforests of the Atherton Tableland, Australia. *Austral Ecol.* **33,** 541–548 (2008).
158. Kardol, P., Bezemer, T. M., Van der Wal, A. & Van der Putten, W. H. Successional trajectories of soil nematode and plant communities in a chronosequence of ex-arable lands. *Biol. Conserv.* **126,** 317–327 (2005).
159. Kariuki, M., Kooyman, R. M., Smith, R. G. B., Wardell-Johnson, G. & Vanclay, J. K. Regeneration changes in tree species abundance, diversity and structure in logged and unlogged subtropical rainforest over a 36-year period. *For. Ecol. Manage.* **236,** 162–176 (2006).
160. Katayama, Y. *et al.* Effects of spilled oil on microbial communities in a tidal flat. *Mar.*

Pollut. Bull. **47,** 85–90 (2003).
161. Kavanagh, R. P. & Stanton, M. A. Bird population recovery 22 years after intensive logging near Eden, New South Wales. *Emu* **103,** 221–231 (2003).
162. Kendrick, G. A. *et al.* Changes in seagrass coverage in Cockburn Sound, Western Australia between 1967 and 1999. *Aquat. Bot.* **73,** 75–87 (2002).
163. Kennard, D. K. Secondary forest succession in a tropical dry forest: patterns of development across a 50-year chronosequence in lowland Bolivia. *J. Trop. Ecol.* **18,** 53–66 (2002).
164. Kil, H. K. & Bae, Y. J. Effects of low-head dam removal on benthic macroinvertebrate communities in a Korean stream. *Animal Cells Syst.* **16,** 69–76 (2012).
165. Kihlman, S. & Kauppila, T. Effects of mining on testate amoebae in a Finnish lake. *J. Paleolimnol.* **47,** 1–15 (2012).
166. Kimball, M. E. & Able, K. W. Nekton utilization of intertidal salt marsh creeks: Tidal influences in natural Spartina, invasive Phragmites, and marshes treated for Phragmites removal. *J. Exp. Mar. Bio. Ecol.* **346,** 87–101 (2007).
167. Kinzie III, R. A., Chong, C., Devrell, J., Lindstrom, D. & Wolff, R. Effects of Water Removal on a Hawaiian Stream Ecosystem 1. *Pacific Sci.* **60,** 1–47 (2006).
168. Klanderud, K. *et al.* Recovery of plant species richness and composition after slash-and-burn agriculture in a tropical rainforest in Madagascar. *Biodivers. Conserv.* **19,** 187–204 (2010).
169. Knops, J. M. H. & Tilman, D. Dynamics of Soil Nitrogen and Carbon Accumulation for 61 Years After Agricultural Abandonment. *Ecology* **81,** 88–98 (2000).
170. Koch, J. M., Grigg, A. H., Gordon, R. K. & Majer, J. D. Arthropods in coarse woody debris in jarrah forest and rehabilitated bauxite mines in Western Australia. *Ann. For. Sci.* **67,** 106 (2010).
171. Konisky, R. A., Burdick, D. M., Dionne, M. & Neckles, H. A. A regional assessment of salt marsh restoration and monitoring in the Gulf of Maine. *Restor. Ecol.* **14,** 516–525 (2006).
172. Konsulova, T. H., Trayanova, A. T. & Todorova, V. R. Sand bank Koketrays - a Case Study on the Effect of Marine Protected Area Designation as a Key approach to Black Sea Biodiversity and Habitat Conservation. *Acta Zool. Bulg.* **61,** 89–97 (2010).
173. Korhola, A. & Blom, T. Marked early 20th century pollution and the subsequent recovery of Töölö Bay, central Helsinki, as indicated by subfossil diatom assemblage changes. *Hydrobiologia* **341,** 169–179 (1996).
174. Koster, D., Lichter, J., Lea, P. D. & Nurse, A. Historical eutrophication in a river estuary complex in mid-coast Maine. *Ecol. Appl.* **17,** 765–778 (2007).
175. Kraufvelin, P., Moy, F. E., Christie, H. & Bokn, T. L. Nutrient addition to experimental rocky shore communities revisited: delayed responses, rapid recovery. *Ecosystems* **9,** 1076–1093 (2006).
176. Krause, J. C., Diesing, M. & Arlt, G. The physical and biological impact of sand extraction: a case study of the western Baltic Sea. *J. Coast. Res.* 215–226 (2010).
177. Kreyling, J., Schmiedinger, A., Macdonald, E. & Beierkuhnlein, C. Slow understory redevelopment after clearcutting in high mountain forests. *Biodivers. Conserv.* **17,** 2339–2355 (2008).
178. Kubach, K. M., Scott, M. C. & Bulak, J. S. Recovery of a temperate riverine fish assemblage from a major diesel oil spill. *Freshw. Biol.* **56,** 503–518 (2011).

179. Kubota, Y., Katsuda, K. & Kikuzawa, K. Secondary succession and effects of clear-logging on diversity in the subtropical forests on Okinawa Island, southern Japan. *Biodivers. Conserv.* **14,** 879–901 (2005).
180. La Peyre, M. K., Gossman, B. & Nyman, J. A. Assessing functional equivalency of nekton habitat in enhanced habitats: comparison of terraced and unterraced marsh ponds. *Estuaries and Coasts* **30,** 526–536 (2007).
181. La Peyre, M. K., Gossman, B. & Piazza, B. P. Short-and long-term response of deteriorating brackish marshes and open-water ponds to sediment enhancement by thin-layer dredge disposal. *Estuaries and Coasts* **32,** 390–402 (2009).
182. Laasonen, P., Muotka, T. & Kivijärvi, I. Recovery of macroinvertebrate communities from stream habitat restoration. *Aquat. Conserv. Mar. Freshw. Ecosyst.* **8,** 101–113 (1998).
183. Lance, B. K., Irons, D. B., Kendall, S. J. & McDonald, L. L. An evaluation of marine bird population trends following the Exxon valdez oil spill, Prince William Sound, Alaska. *Mar. Pollut. Bull.* **42,** 298–309 (2001).
184. Lappalainen, A. & Pesonen, L. Changes in fish community structure after cessation of waste water discharge in a coastal bay area west of Helsinki, northern Baltic Sea. *Arch. Fish. Mar. Res. fur Fischerei und Meeresforsch.* **48,** 226–241 (2000).
185. Lardicci, C., Como, S., Corti, S. & Rossi, F. Changes and recovery of macrozoobenthic communities after restoration measures of the Orbetello Lagoon (Tyrrhenian coast, Italy). *Aquat. Conserv. Mar. Freshw. Ecosyst.* **11,** 281–287 (2001).
186. Laughlin, D. C. *et al.* Assessing targets for the restoration of herbaceous vegetation in ponderosa pine forests. *Restor. Ecol.* **14,** 548–560 (2006).
187. Lawrence, D. & Foster, D. Changes in forest biomass, litter dynamics and soils following shifting cultivation in southern Mexico: An overview. *Interciencia* **27,** 400–+ (2002).
188. Lebrija-Trejos, E., Bongers, F., Pérez-García, E. A. & Meave, J. A. Successional Change and Resilience of a Very Dry Tropical Deciduous Forest Following Shifting Agriculture. *Biotropica* **40,** 422–431 (2008).
189. Lenihan, H. S. *et al.* Cascading of habitat degradation: oyster reefs invaded by refugee fishes escaping stress. *Ecol. Appl.* **11,** 764–782 (2001).
190. Lepori, F., Palm, D., Brännäs, E. & Malmqvist, B. Does restoration of structural heterogeneity in streams enhance fish and macroinvertebrate diversity? *Ecol. Appl.* **15,** 2060–2071 (2005).
191. Lepori, F., Palm, D. & Malmqvist, B. Effects of stream restoration on ecosystem functioning: detritus retentiveness and decomposition. *J. Appl. Ecol.* **42,** 228–238 (2005).
192. Levine, S. N. & Schindler, D. W. Phosphorus, nitrogen, and carbon dynamics of Experimental Lake 303 during recovery from eutrophication. *Can. J. Fish. Aquat. Sci.* **46,** 2–10 (1989).
193. Levine, S. N. *et al.* The eutrophication of Lake Champlain's northeastern arm: Insights from paleolimnological analyses. *J. Great Lakes Res.* **38,** 35–48 (2012).
194. Lewis, R. R. & Gilmore, R. G. Important considerations to achieve successful mangrove forest restoration with optimum fish habitat. *Bull. Mar. Sci.* **80,** 823–837 (2007).
195. Li, W., Zhang, Z. & Jeppesen, E. The response of Vallisneria spinulosa (Hydrocharitaceae) to different loadings of ammonia and nitrate at moderate phosphorus concentration: a mesocosm approach. *Freshw. Biol.* **53,** 2321–2330 (2008).
196. Lima, A. *et al.* Nitrogen-fixing bacteria communities occurring in soils under different

uses in the Western Amazon Region as indicated by nodulation of siratro (<i>Macroptilium atropurpureum</i>). *Plant Soil* **319,** 127–145 (2009).
197. Lin, T. C. *et al.* Typhoon Disturbance and Forest Dynamics: Lessons from a Northwest Pacific Subtropical Forest. *Ecosystems* **14,** 127–143 (2011).
198. Little, J. L., Hall, R. I., Quinlan, R. & Smol, J. P. Past trophic status and hypolimnetic anoxia during eutrophicaton and remediation of Gravenhurst Bay, Ontario: comparison of diatoms, chironomids, and historical records. *Can. J. Fish. Aquat. Sci.* **57,** 333–341 (2000).
199. Long, D. T. *et al.* Assessing the response of watersheds to catastrophic (logging) and possible secular (global temperature change) perturbations using sediment-chemical chronologies. *Appl. Geochemistry* **25,** 143–158 (2010).
200. Louette, G., Declerck, S., Vandekerkhove, J. & De Meester, L. Evaluation of restoration measures in a shallow lake through a comparison of present day zooplankton communities with historical samples. *Restor. Ecol.* **17,** 629–640 (2009).
201. Louette, G., Declerck, S., Vandekerkhove, J. & De Meester, L. Evaluation of restoration measures in a shallow lake through a comparison of present day zooplankton communities with historical samples. *Restor. Ecol.* **17,** 629–640 (2009).
202. Louhi, P. *et al.* Twenty years of stream restoration in Finland: little response by benthic macroinvertebrate communities. *Ecol. Appl.* **21,** 1950–1961 (2011).
203. Loya, D. T. & Jules, E. S. Use of species richness estimators improves evaluation of understory plant response to logging: a study of redwood forests. *Plant Ecol.* **194,** 179–194 (2008).
204. Lu, L. & Wu, R. S. S. A field experimental study on recolonization and succession of macrobenthic infauna in defaunated sediment contaminated with petroleum hydrocarbons. *Estuar. Coast. Shelf Sci.* **68,** 627–634 (2006).
205. Lytle, D. A. & Peckarsky, B. L. Spatial and temporal impacts of a diesel fuel spill on stream invertebrates. *Freshw. Biol.* **46,** 693–704 (2001).
206. Macedo, M. O. *et al.* Changes in soil C and N stocks and nutrient dynamics 13 years after recovery of degraded land using leguminous nitrogen-fixing trees. *For. Ecol. Manage.* **255,** 1516–1524 (2008).
207. Madgwick, G. *et al.* Centennial- scale changes to the aquatic vegetation structure of a shallow eutrophic lake and implications for restoration. *Freshw. Biol.* **56,** 2620–2636 (2011).
208. Mäkiranta, P., Riutta, T., Penttilä, T. & Minkkinen, K. Dynamics of net ecosystem CO 2 exchange and heterotrophic soil respiration following clearfelling in a drained peatland forest. *Agric. For. Meteorol.* **150,** 1585–1596 (2010).
209. Mallin, M. A. *et al.* Hurricane Effects on Water Quality and Benthos in the Cape Fear Watershed : Natural and Anthropogenic Impacts. *Ecol. Appl.* **9,** 350–362 (2009).
210. Marchetto, A. *et al.* Lake Maggiore (N. Italy) trophic history: fossil diatom, plant pigments, and chironomids, and comparison with long-term limnological data. *Quat. Int.* **113,** 97–110 (2004).
211. Marin-Spiotta, E., Ostertag, R. & Silver, W. L. Long-term patterns in tropical reforestation: Plant community composition and aboveground biomass accumulation. *Ecol. Appl.* **17,** 828–839 (2007).
212. Marques, L. *et al.* Response of intertidal macrobenthic communities and primary producers to mitigation measures in a temperate estuary. *Ecol. Indic.* **25,** 10–22 (2013).
213. Martay, B., Hughes, F. & Doberski, J. A comparison of created and ancient fenland using ground beetles as a measure of conservation value. *Insect Conserv. Divers.* **5,** 251–263

(2012).
214. Massimino, D., Masin, S., Bani, L., Dranzoa, C. & Massa, R. Partial recovery of an African rainforest bird community 35 years after logging. *Ethol. Ecol. Evol.* **20,** 391–399 (2008).
215. Mathieu, J. *et al.* Recovery of Soil Macrofauna Communities after Forest Clearance in Eastern Amazonia, Brazil. *Conserv. Biol.* **19,** 1598–1605 (2005).
216. Matkin, C. O., Saulitis, E. L., Ellis, G. M., Olesiuk, P. & Rice, S. D. Ongoing population-level impacts on killer whales Orcinus orca following the 'Exxon Valdez'oil spill in Prince William Sound, Alaska. *Mar. Ecol. Prog. Ser.* **356,** 269–281 (2008).
217. Mazzei, L. *et al.* Above-ground biomass dynamics after reduced-impact logging in the Eastern Amazon. *For. Ecol. Manage.* **259,** 367–373 (2010).
218. McCall, B. D. & Pennings, S. C. Disturbance and recovery of salt marsh arthropod communities following BP Deepwater Horizon oil spill. *PLoS One* **7,** e32735 (2012).
219. McKinley, V. L. & Wolek, R. The effects of tallgrass prairie restoration on soil quality indicators. in *Proc. 18th N. Am. Prairie Conference* **138,** (2003).
220. McLauchlan, K. K., Craine, J. M., Oswald, W. W., Leavitt, P. R. & Likens, G. E. Changes in nitrogen cycling during the past century in a northern hardwood forest. *Proc. Natl. Acad. Sci.* **104,** 7466–7470 (2007).
221. McPherson, T. S. & Timmer, V. R. Amelioration of degraded soils under red pine plantations on the Oak Ridges Moraine, Ontario. *Can. J. Soil Sci.* **82,** 375–388 (2002).
222. Melville, F., Andersen, L. E. & Jolley, D. F. The Gladstone (Australia) oil spill–Impacts on intertidal areas: Baseline and six months post-spill. *Mar. Pollut. Bull.* **58,** 263–271 (2009).
223. Meriläinen, J. J., Hynynen, J., Palomäki, A., Mäntykoski, K. & Witick, A. Environmental history of an urban lake: a palaeolimnological study of Lake Jyväsjärvi, Finland. *J. Paleolimnol.* **30,** 387–406 (2003).
224. Michelutti, N., Douglas, M. S. V & Smol, J. P. Tracking recent recovery from eutrophication in a high arctic lake (Meretta Lake, Cornwallis Island, Nunavut, Canada) using fossil diatom assemblages. *J. Paleolimnol.* **28,** 377–381 (2002).
225. Michelutti, N., Hermanson, M. H., Smol, J. P., Dillon, P. J. & Douglas, M. S. V. Delayed response of diatom assemblages to sewage inputs in an Arctic lake. *Aquat. Sci.* **69,** 523–533 (2007).
226. Middleton, B., Devlin, D., Proffitt, E., McKee, K. & Cretini, E. F. Characteristics of mangrove swamps managed for mosquito control in eastern Florida, USA. *Mar. Ecol. Prog. Ser.* **371,** 117–129 (2008).
227. Middleton, E. L., Bever, J. D. & Schultz, P. A. The effect of restoration methods on the quality of the restoration and resistance to invasion by exotics. *Restor. Ecol.* **18,** 181–187 (2010).
228. Miller, T. E., Gornish, E. S. & Buckley, H. L. Climate and coastal dune vegetation: Disturbance, recovery, and succession. *Plant Ecol.* **206,** 97–104 (2009).
229. Montagna, P. A., Kalke, R. D. & Ritter, C. Effect of restored freshwater inflow on macrofauna and meiofauna in upper Rincon Bayou, Texas, USA. *Estuaries* **25,** 1436–1447 (2002).
230. Moola, F. M. & Vasseur, L. Recovery of late-seral vascular plants in a chronosequence of post-clearcut forest stands in coastal Nova Scotia, Canada. *Vegetatio* **172,** 183–197 (2004).
231. Moss, B. *et al.* Consequences of reduced nutrient loading on a lake system in a lowland catchment: deviations from the norm? *Freshw. Biol.* **50,** 1687–1705 (2005).

232. Moynahan, O. S., Zabinski, C. A. & Gannon, J. E. Microbial Community Structure and Carbon- Utilization Diversity in a Mine Tailings Revegetation Study. *Restor. Ecol.* **10,** 77–87 (2002).
233. Mueller, a. D. *et al.* Recovery of the forest ecosystem in the tropical lowlands of northern Guatemala after disintegration of Classic Maya polities. *Geology* **38,** 523–526 (2010).
234. Munkes, B. Eutrophication, phase shift, the delay and the potential return in the Greifswalder Bodden, Baltic Sea. *Aquat. Sci.* **67,** 372–381 (2005).
235. Ndour, N. Y. B. *et al.* Characteristics of microbial habitats in a tropical soil subject to different fallow management. *Appl. Soil Ecol.* **38,** 51–61 (2008).
236. Negrete-Yankelevich, S., Fragoso, C., Newton, A. C. & Heal, O. W. Successional changes in soil, litter and macroinvertebrate parameters following selective logging in a Mexican Cloud Forest. *Appl. Soil Ecol.* **35,** 340–355 (2007).
237. Nikitik, C. C. S. & Robinson, A. W. Patterns in benthic populations in the Milford Haven waterway following the [`]Sea Empress' oil spill with special reference to amphipods. *Mar. Pollut. Bull.* **46,** 1125–1141 (2003).
238. Nordi, G. A., Glud, R. N., Gaard, E. & Simonsen, K. Environmental impacts of coastal fish farming; Carbon and Nitrogen budgets for trout farming in Kaldbacksfjord, Faroe Islands. *Mar. Ecol. Ser.* (2011).
239. Norman, M. A., Koch, J. M., Grant, C. D., Morald, T. K. & Ward, S. C. Vegetation succession after bauxite mining in Western Australia. *Restor. Ecol.* **14,** 278–288 (2006).
240. Obayori, O. S., Ilori, M. O., Adebusoye, S. A., Amund, O. O. & Oyetibo, G. O. Microbial population changes in tropical agricultural soil experimentally contaminated with crude petroleum. *African J. Biotechnol.* **7,** (2008).
241. O'Brien, S. L. & Jastrow, J. D. Physical and chemical protection in hierarchical soil aggregates regulates soil carbon and nitrogen recovery in restored perennial grasslands. *Soil Biol. Biochem.* **61,** 1–13 (2013).
242. O'Brien, W. J. *et al.* Long-term response and recovery to nutrient addition of a partitioned arctic lake. *Freshw. Biol.* **50,** 731–741 (2005).
243. Ocon, C. S., Capítulo, A. R. & Paggi, A. C. Evaluation of zoobenthic assemblages and recovery following petroleum spill in a coastal area of Río de la Plata estuarine system, South America. *Environ. Pollut.* **156,** 82–89 (2008).
244. O'Dwyer, B., Crockford, L., Jordan, P., Hislop, L. & Taylor, D. A palaeolimnological investigation into nutrient impact and recovery in an agricultural catchment. *J. Environ. Manage.* **124,** 147–155 (2013).
245. Orabi, G., Moir, M. L. & Majer, J. D. Assessing the success of mine restoration using Hemiptera as indicators. *Aust. J. Zool.* **58,** 243–249 (2010).
246. Osborne, K., Dolman, A. M., Burgess, S. C. & Johns, K. A. Disturbance and the dynamics of coral cover on the Great Barrier Reef (1995‚Äì2009). *PLoS One* **6,** e17516 (2011).
247. Ostertag, R., Marín-Spiotta, E., Silver, W. & Schulten, J. Litterfall and Decomposition in Relation to Soil Carbon Pools Along a Secondary Forest Chronosequence in Puerto Rico. *Ecosystems* **11,** 701–714 (2008).
248. O'Sullivan, O. S., Horswill, P., Phoenix, G. K., Lee, J. A. & Leake, J. R. Recovery of soil nitrogen pools in species- rich grasslands after 12 years of simulated pollutant nitrogen deposition: a 6- year experimental analysis. *Glob. Chang. Biol.* **17,** 2615–2628 (2011).

249. Ottonetti, L., Tucci, L. & Santini, G. Recolonization patterns of ants in a rehabilitated lignite mine in central Italy: potential for the use of Mediterranean ants as indicators of restoration processes. *Restor. Ecol.* **14,** 60–66 (2006).
250. Ouédraogo, D.-Y. *et al.* Thinning after selective logging facilitates floristic composition recovery in a tropical rain forest of Central Africa. *For. Ecol. Manage.* **262,** 2176–2186 (2011).
251. Owiunji, I. & Plumptre, A. J. Bird communities in logged and unlogged compartments in Budongo Forest, Uganda. *For. Ecol. Manage.* **108,** 115–126 (1998).
252. Pais, M. P. & Varanda, E. M. Arthropod recolonization in the restoration of a semideciduous forest in southeastern Brazil. *Neotrop. Entomol.* **39,** 198–206 (2010).
253. Paling, E. I., Kobryn, H. T. & Humphreys, G. Assessing the extent of mangrove change caused by Cyclone Vance in the eastern Exmouth Gulf, northwestern Australia. *Estuar. Coast. Shelf Sci.* **77,** 603–613 (2008).
254. Palladini, J. D., Jones, M. G., Sanders, N. J. & Jules, E. S. The recovery of ant communities in regenerating temperate conifer forests. *For. Ecol. Manage.* **242,** 619–624 (2007).
255. Pérez-del-Olmo, A., Montero, F. E., Raga, J. A., Fernández, M. & Kostadinova, A. Follow-up trends of parasite community alteration in a marine fish after the Prestige oil-spill: Shifting baselines? *Environ. Pollut.* **157,** 221–228 (2009).
256. Persson, L. *et al.* Culling prey promotes predator recovery - alternative states in a whole-lake experiment. *Science* **316,** 1743–1746 (2007).
257. Piazza, B. P. & La Peyre, M. K. The effect of Hurricane Katrina on nekton communities in the tidal freshwater marshes of Breton Sound, Louisiana, USA. *Estuar. Coast. Shelf Sci.* **83,** 97–104 (2009).
258. Pinard, M. A., Barker, M. G. & Tay, J. Soil disturbance and post-logging forest recovery on bulldozer paths in Sabah, Malaysia. *For. Ecol. Manage.* **130,** 213–225 (2000).
259. Poirrier, M. A., Del Rey, Z. R. & Spalding, E. A. Acute disturbance of lake pontchartrain benthic communities by Hurricane Katrina. *Estuar. Coast.* **31,** 1221–1228 (2008).
260. Pollard, A. I. & Reed, T. Benthic invertebrate assemblage change following dam removal in a Wisconsin stream. *Hydrobiologia* **513,** 51–58 (2004).
261. Power, S. A., Green, E. R., Barker, C. G., BELL, J. N. B. & Ashmore, M. R. Ecosystem recovery: heathland response to a reduction in nitrogen deposition. *Glob. Chang. Biol.* **12,** 1241–1252 (2006).
262. Prat, N. *et al.* Effect of dumping and cleaning activities on the aquatic ecosystems of the Guadiamar River following a toxic flood. *Sci. Total Environ.* **242,** 231–248 (1999).
263. Preen, A. R., Lee Long, W. J. & Coles, R. G. Flood and cyclone related loss, and partial recovery, of more than 1000 km2 of seagrass in Hervey Bay, Queensland, Australia. *Aquat. Bot.* **52,** 3–17 (1995).
264. Quintero, I. & Roslin, T. Rapid recovery of dung beetle communities following habitat fragmentation in Central Amazonia. *Ecology* **86,** 3303–3311 (2005).
265. Rab, M. A. Recovery of soil physical properties from compaction and soil profile disturbance caused by logging of native forest in Victorian Central Highlands, Australia. *For. Ecol. Manage.* **191,** 329–340 (2004).
266. Raman, T. R. S. Effect of slash-and-burn shifting cultivation on rainforest birds in Mizoram, northeast India. *Conserv. Biol.* **15,** 685–698 (2001).

267. Ramírez-Barajas, P. J., Islebe, G. A. & Calmé, S. Impact of Hurricane Dean (2007) on Game Species of the Selva Maya, Mexico. *Biotropica* **44,** 402–411 (2012).
268. Ramsey, E. . *et al.* Daily MODIS data trends of hurricane-induced forest impact and early recovery. *Photogramm. Eng. Remote Sensing* **77,** 1133–1143 (2011).
269. Raynor, E. J., Pierce, A. R., Owen, T. M., Leumas, C. M. & Rohwer, F. C. Short-term demographic responses of a coastal waterbird community after two major hurricanes. *Waterbirds* **36,** 88–93 (2013).
270. Read, L. & Lawrence, D. Litter nutrient dynamics during succession in dry tropical forests of the Yucatan: Regional and seasonal effects. *Ecosystems* **6,** 747–761 (2003).
271. Reay, S. D. & Norton, D. a. Assessing the Success of Restoration Plantings in a Temperate New Zealand Forest. *Restor. Ecol.* **7,** 298–308 (1999).
272. Redi, B. H., Van Aarde, R. J. & Wassenaar, T. D. Coastal dune forest development and the regeneration of millipede communities. *Restor. Ecol.* **13,** 284–291 (2005).
273. Relini, G., Relini, M., Torchia, G. & Palandri, G. Ten years of censuses of fish fauna on the Loano artificial reef. *ICES J. Mar. Sci. J. du Cons.* **59,** S132–S137 (2002).
274. Rhemtulla, J. M., Mladenoff, D. J. & Clayton, M. K. Historical forest baselines reveal potential for continued carbon sequestration. *Proc. Natl. Acad. Sci.* **106,** 6082–6087 (2009).
275. Rietsma, C. S., Monteiro, R. O. & Valiela, I. Plant cover, herbivory, and resiliency in a Cape Cod salt marsh: Multi-year responses and recovery following manipulation of nutrients and competition. *Estuaries and Coasts* **34,** 198–210 (2011).
276. Rogers, K. M. Stable carbon and nitrogen isotope signatures indicate recovery of marine biota from sewage pollution at Moa Point, New Zealand. *Mar. Pollut. Bull.* **46,** 821–827 (2003).
277. Roldan, A., Garcia, C. & Albaladejo, J. AM fungal abundance and activity in a chronosequence of abandoned fields in a semiarid Mediterranean site. *Arid Soil Res. Rehabil.* **11,** 211–220 (1997).
278. Roman, C. T., Raposa, K. B., Adamowicz, S. C., James- Pirri, M. & Catena, J. G. Quantifying vegetation and nekton response to tidal restoration of a New England salt marsh. *Restor. Ecol.* **10,** 450–460 (2002).
279. Rossouw, J., Van Rensburg, L., Claassens, S. & van Rensburg, P. J. J. Nematodes as indicators of ecosystem development during platinum mine tailings reclamation. *Environmentalist* **28,** 99–107 (2008).
280. Rublee, P. A. & Bettez, N. D. in *Rotifera IX* 229–232 (Springer, 2001).
281. Rufaut, C. G. & Craw, D. Geoecology of ecosystem recovery at an inactive coal mine site, New Zealand. *Environ. Earth Sci.* **60,** 1425–1437 (2010).
282. Sabater, S. Diatom communities as indicators of environmental stress in the Guadiamar River, SW. Spain, following a major mine tailings spill. *J. Appl. Phycol.* **12,** 113–124 (2000).
283. Sadaba, R. B. & Sarinas, B. G. S. Fungal communities in bunker C oil-impacted sites off southern Guimaras, Philippines: a post-spill assessment of Solar 1 oil spill. *Bot. Mar.* **53,** 565–575 (2010).
284. Samways, M. J., Sharratt, N. J. & Simaika, J. P. Effect of alien riparian vegetation and its removal on a highly endemic river macroinvertebrate community. *Biol. Invasions* **13,** 1305–1324 (2011).
285. Sand- Jensen, K. *et al.* 100 years of vegetation decline and recovery in Lake Fure, Denmark. *J. Ecol.* **96,** 260–271 (2008).
286. Sandman, O. *et al.* Short-code paleolimnological investigation of Lake Pihlajavesi in the

Saimaa Lake complex, eastern Finalnd: assessment of habitat quality of an endemic and endangered seal population. *J. Paleolimnol.* **24,** 317–329 (2000).
287. Sato, T. *et al.* Temporal dynamics and resilience of fine litterfall in relation to typhoon disturbances over 14 years in an old-growth lucidophyllous forest in southwestern Japan. *Plant Ecol.* **208,** 187–198 (2010).
288. Savage, C., Elmgren, R. & Larsson, U. Effects of sewage-derived nutrients on an estuarine macrobenthic community. *Mar. Ecol. Prog. Ser.* **243,** 67–82 (2002).
289. Scalley, T. H., Scatena, F. N. & Estrada-Ruiz, C. R. Changes in Structure, Composition, and Nutrients During 15 Years of Hurricane-induced Succession in a Subtropical Wet Forest in Puerto Rico. *Biotropica* **42,** 455–463 (2010).
290. Schwenke, G. D., Mulligan, D. R. & Bell, L. C. Soil stripping and replacement for the rehabilitation of bauxite-mined land at Weipa. I. Initial changes to soil organic matter and related parameters. *Soil Res.* **38,** 345–370 (2000).
291. Scopélitis, J. *et al.* Changes of coral communities over 35 years: Integrating in situ and remote-sensing data on Saint-Leu Reef (la Reunion, Indian Ocean). *Estuar. Coast. Shelf Sci.* **84,** 342–352 (2009).
292. Scowcroft, P. G. & Jeffrey, J. Potential significance of frost, topographic relief, and Acacia koa stands to restoration of mesic Hawaiian forests on abandoned rangeland. *For. Ecol. Manage.* **114,** 447–458 (1999).
293. Seo, K. W. *et al.* Seedling Growth and Heavy Metal Accumulation of Candidate Woody Species for Revegetating Korean Mine Spoils. *Restor. Ecol.* **16,** 702–712 (2008).
294. Serieyssol, C. A., Edlund, M. B. & Kallemeyn, L. W. Impacts of settlement, damming, and hydromanagement in two boreal lakes: a comparative paleolimnological study. *J. Paleolimnol.* **42,** 497–513 (2009).
295. Simmonds, S. J., Majer, J. D. & Nichols, O. G. A comparative study of spider (Araneae) communities of rehabilitated bauxite mines and surrounding forest in the southwest of Western Australia. *Restor. Ecol.* **2,** 247–260 (1994).
296. Simon, M. L., Cherry, D. S., Currie, R. J. & Zipper, C. E. The ecotoxicological recovery of Ely Creek and tributaries (Lee County, VA) after remediation of acid mine drainage. *Environ. Monit. Assess.* **184,** 2559–2574 (2012).
297. Simpson, A. W. & Watling, L. An investigation of the cumulative impacts of shrimp trawling on mud-bottom fishing grounds in the Gulf of Maine: effects on habitat and macrofaunal community structure. *ICES J. Mar. Sci.* **63,** 1616–1630 (2006).
298. Skalski, J. R., Coats, D. A. & Fukuyama, A. K. Criteria for oil spill recovery: a case study of the intertidal community of Prince William Sound, Alaska, following the Exxon Valdez oil spill. *Environ. Manage.* **28,** 9–18 (2001).
299. Slate, J. E. & Stevenson, R. J. Recent and abrupt environmental change in the Florida everglades indicated from siliceous microfossils. *Wetlands* **20,** 346–356 (2000).
300. Smith, S. D. A. & Simpson, R. D. Recovery of benthic communities at Macquarie Island (sub-Antarctic) following a small oil spill. *Mar. Biol.* **131,** 567–581 (1998).
301. Sparks-McConkey, P. J. & Watling, L. Effects on the ecological integrity of a soft-bottom habitat from a trawling disturbance. *Hydrobiologia* **456,** 73–85 (2001).
302. Stone, M. K. & Wallace, J. B. Long-term recovery of a mountain stream from clearcut logging: the effects of forest succession on benthic invertebrate community structure. *Freshw. Biol.* **39,** 151–169 (1998).
303. Sublette, K. *et al.* Monitoring soil ecosystem recovery following bioremediation of a

terrestrial crude oil spill with and without a fertilizer amendment. *Soil Sediment Contam.* **16,** 181–208 (2007).
304. Tallman, J. C. & Forrester, G. E. Oyster grow-out cages function as artificial reefs for temperate fishes. *Trans. Am. Fish. Soc.* **136,** 790–799 (2007).
305. Tam, T.-W. & Ang, P. O. Repeated physical disturbances and the stability of sub-tropical coral communities in Hong Kong, China. *Aquat. Conserv. Mar. Freshw. Ecosyst.* **18,** 1005–1024 (2008).
306. Teal, J. M. & Peterson, S. Restoration benefits in a watershed context. *J. Coast. Res.* 132–140 (2005).
307. Teels, B. M., Rewa, C. A. & Myers, J. Aquatic condition response to riparian buffer establishment. *Wildl. Soc. Bull.* **34,** 927–935 (2006).
308. Teh, Y. A., Silver, W. L. & Scatena, F. N. A decade of belowground reorganization following multiple disturbances in a subtropical wet forest. *Plant Soil* **323,** 197–212 (2009).
309. Templer, P. H., Groffman, P. M., Flecker, A. S. & Power, A. G. Land use change and soil nutrient transformations in the Los Haitises region of the Dominican Republic. *Soil Biol. Biochem.* **37,** 215–225 (2005).
310. Thompson, G. G. & Thompson, S. A. Mammals or reptiles, as surveyed by pit-traps, as bio-indicators of rehabilitation success for mine sites in the goldfields region of Western Australia? *Pacific Conserv. Biol.* **11,** 268–286 (2005).
311. Tuck, I. D., Hall, S. J., Robertson, M. R., Armstrong, E. & Basford, D. J. Effects of physical trawling disturbance in a previously unfished sheltered Scottish sea loch. *Mar. Ecol. Prog. Ser.* **162,** 227–242 (1998).
312. Valiela, I. *et al.* Ecological Effects of Major Storms on Coastal Watersheds and Coastal Waters: Hurricane Bob on Cape Cod. *Source J. Coast. Res. J. Coast. Res.* **14,** 218–238 (1998).
313. Valtonen, E. T., Holmes, J. C., Aronen, J. & Rautalahti, I. Parasite communities as indicators of recovery from pollution: parasites of roach (Rutilus rutilus) and perch (Perca fluviatilis) in Central Finland. *Parasitology* **126,** S43–S52 (2003).
314. van Aarde, R. J. *et al.* An Evaluation of Habitat Rehabilitation on Coastal Dune Forests in Northern KwaZulu- Natal, South Africa. *Restor. Ecol.* **4,** 334–345 (1996).
315. Van Do, T., Osawa, A. & Thang, N. T. Recovery process of a mountain forest after shifting cultivation in Northwestern Vietnam. *For. Ecol. Manage.* **259,** 1650–1659 (2010).
316. van Kleef, H. H. *et al.* Effects of reduced nitrogen and sulphur deposition on the water chemistry of moorland pools. *Environ. Pollut.* **158,** 2679–2685 (2010).
317. Vargas, R. How a hurricane disturbance influences extreme CO 2 fluxes and variance in a tropical forest. *Environ. Res. Lett.* **7,** 35704 (2012).
318. Vargas, R., Hasselquist, N., Allen, E. B. & Allen, M. F. Effects of a hurricane disturbance on aboveground forest structure, arbuscular mycorrhizae and belowground carbon in a restored tropical forest. *Ecosystems* **13,** 118–128 (2010).
319. Vaudrey, J. M. P., Kremer, J. N., Branco, B. F. & Short, F. T. Eelgrass recovery after nutrient enrichment reversal. *Aquat. Bot.* **93,** 237–243 (2010).
320. Vedrova, E. F., Mukhortova, L. V, Ivanov, V. V, Krivobokov, L. V & Boloneva, M. V. Post-logging organic matter recovery in forest ecosystems of eastern Baikal region. *Biol. Bull.* **37,** 69–79 (2010).
321. Vieira, D. L. M., Scariot, A., Sampaio, A. B. & Holl, K. D. Tropical dry-forest regeneration from root suckers in Central Brazil. *J. Trop. Ecol.* **22,** 353–357 (2006).

322. Viljanen, M., Drabkova, V., Avinsky, V., Kapustina, L. & Raspletina, G. Ecological state and monitoring of limnological and biological parameters in Lake Ladoga, Russia. *Aquat. Ecosyst. Health Manag.* **11,** 61–74 (2008).
323. Villa-Galaviz, E., Boege, K. & del-Val, E. Resilience in Plant-Herbivore Networks during Secondary Succession. *PLoS One* **7,** e53009 (2012).
324. Waddington, J. M. & Warner, K. D. Atmospheric CO2 sequestration in restored mined peatlands. *Ecoscience* **8,** 359 (2001).
325. Walker, S., Wilson, J. B. & Lee, W. G. Recovery of short tussock and woody species guilds in ungrazed Festuca novae-zelandiae short tussock grassland with fertiliser or irrigation. *N. Z. J. Ecol.* **27,** 179–189 (2003).
326. Walter, C. A., Nelson, D. & Earle, J. I. Assessment of Stream Restoration: Sources of Variation in Macroinvertebrate Recovery throughout an 11- Year Study of Coal Mine Drainage Treatment. *Restor. Ecol.* **20,** 431–440 (2012).
327. Walton, M. E., Le Vay, L., Lebata, J. H., Binas, J. & Primavera, J. H. Assessment of the effectiveness of mangrove rehabilitation using exploited and non-exploited indicator species. *Biol. Conserv.* **138,** 180–188 (2007).
328. Wang, C. M. *et al.* Soil carbon changes following afforestation with Olga Bay larch (Larix olgensis Henry) in northeastern China. *J. Integr. Plant Biol.* **48,** 503–512 (2006).
329. Wangpakapattanawong, P., Kavinchan, N., Vaidhayakarn, C., Schmidt-Vogt, D. & Elliott, S. Fallow to forest: applying indigenous and scientific knowledge of swidden cultivation to tropical forest restoration. *For. Ecol. Manage.* **260,** 1399–1406 (2010).
330. Ward, S. C. Soil development on rehabilitated bauxite mines in south-west Australia. *Soil Res.* **38,** 453–464 (2000).
331. Watts, C. H., Clarkson, B. R. & Didham, R. K. Rapid beetle community convergence following experimental habitat restoration in a mined peat bog. *Biol. Conserv.* **141,** 568–579 (2008).
332. Watts, C. H., Vojvodic-Vukovic, M., Arnold, G. C. & Didham, R. K. A comparison of restoration techniques to accelerate recovery of litter decomposition and microbial activity in an experimental peat bog restoration trial. *Wetl. Ecol. Manag.* **16,** 199–217 (2008).
333. Weckström, K. Assessing recent eutrophication in coastal waters of the Gulf of Finland (Baltic Sea) using subfossil diatoms. *J. Paleolimnol.* **35,** 571–592 (2006).
334. Whitford, W. G., De Soyza, A. G., Van Zee, J. W., Herrick, J. E. & Havstad, K. M. Vegetation, soil, and animal indicators of rangeland health. *Environ. Monit. Assess.* **51,** 179–200 (1998).
335. Wiens, J. A., Day, R. H., Murphy, S. M. & Parker, K. R. Changing habitat and habitat use by birds after the Exxon Valdez oil spill, 1989-2001. *Ecol. Appl.* **14,** 1806–1825 (2004).
336. Willett, T. R. Spiders and other arthropods as indicators in old-growth versus logged redwood stands. *Restor. Ecol.* **9,** 410–420 (2001).
337. Williams, D. E. & Miller, M. W. Attributing mortality among drivers of population decline in Acropora palmata in the Florida Keys (USA). *Coral Reefs* **31,** 369–382 (2012).
338. Williams, A. *et al.* Seamount megabenthic assemblages fail to recover from trawling impacts. *Mar. Ecol.* **31,** 183–199 (2010).
339. Williams, M. R. *et al.* Recovery of bird populations after clearfelling of tall open eucalypt forest in Western Australia. *J. Appl. Ecol.* **38,** 910–920 (2001).
340. Williams, M. *et al.* Carbon sequestration and biodiversity of re-growing miombo woodlands in Mozambique. *For. Ecol. Manage.* **254,** 145–155 (2008).

341. Winfield, I. J. *et al.* Changes in the fish community of Loch Leven: untangling anthropogenic pressures. *Hydrobiologia* **681,** 73–84 (2012).
342. Wolinski, A. L. T. O., Lana, P. C. & Sandrini-Neto, L. Is the cutting of oil contaminated marshes an efficient clean-up technique in a subtropical estuary? *Mar. Pollut. Bull.* **62,** 1227–1232 (2011).
343. Yanai, R. D. The effect of whole-tree harvest on phosphorus cycling in a northern hardwood forest. *For. Ecol. Manage.* **104,** 281–295 (1998).
344. Yin, B., Crowley, D., Sparovek, G., De Melo, W. J. & Borneman, J. Bacterial functional redundancy along a soil reclamation gradient. *Appl. Environ. Microbiol.* **66,** 4361–4365 (2000).
345. Zacheis, A. & Doran, K. Resistance and resilience of floating mat fens in interior Alaska following airboat disturbance. *Wetlands* **29,** 236–247 (2009).
346. Zaloumis, N. P. & Bond, W. J. Grassland restoration after afforestation: No direction home? *Austral Ecol.* **36,** 357–366 (2011).
347. Zanne, A. E. & Chapman, C. A. Expediting reforestation in tropical grasslands: distance and isolation from seed sources in plantations. *Ecol. Appl.* **11,** 1610–1621 (2001).
348. Zenetos, A. *et al.* The Eurobulker oil spill: mid-term changes of some ecosystem indicators. *Mar. Pollut. Bull.* **48,** 122–131 (2004).
